# ACTIV-6: Operationalizing a decentralized, outpatient randomized platform trial to evaluate efficacy of repurposed medicines for COVID-19

**DOI:** 10.1017/cts.2023.644

**Published:** 2023-10-31

**Authors:** The Accelerating Covid-19 Therapeutic Interventions and Vaccines (ACTIV)-6 Study Group

**Keywords:** Decentralized trial, Study design, platform trial, placebo-controlled trial, randomized clinical trial

## Abstract

Despite the availability of vaccinations, severe acute respiratory syndrome coronavirus 2 (SARS-CoV-2) continues to cause Coronavirus Disease 2019 (COVID-19) infection with a spectrum of disease in the acute setting. Transmission, infection, and severe disease remain common. There is a critical need to establish treatment regimens in the ambulatory setting that can reduce symptom burden and potentially prevent progression to severe disease and death. Many existing medicines previously approved for other uses may have benefit but remain unproven in informative clinical trials.

Accelerating COVID-19 Therapeutic Interventions and Vaccines (ACTIV)-6 is a decentralized, placebo-controlled, double-blind, randomized, platform trial that has now enrolled more than 7500 participants and has reported on the effectiveness of ivermectin at two doses, fluticasone, and fluvoxamine for helping people with COVID-19. With additional repurposed therapies added to the platform, ACTIV-6 continues to enroll symptomatic outpatients aged ≥ 30 years with a confirmed positive PCR or antigen test for SARS-CoV-2. Potential participants are screened and enrolled online, through a call center, or facilitated by local study sites. Participants consent electronically and are randomized to placebo or to one of the open study drugs for which they are eligible at the time of enrollment. A shared, contemporary placebo approach is used. Participants receive study drug in the mail and remain on study for up to 180 days. While enrolled, electronic patient-reported outcome assessments are used to monitor symptoms, healthcare utilization, and mortality. The primary endpoint is time to recovery or a composite of hospitalization and mortality within 28 days. Symptoms, acute healthcare utilization, and the Patient-Reported Outcomes Measurement Information System-29 are collected for up to 180 days.

Using a decentralized trial approach allowed the ACTIV-6 platform to increase both reach and rate of enrollment. The decentralized approach did not simplify regulatory oversight, and we found unanticipated challenges in patient behavior and the study drug delivery process. Despite challenges, ACTIV-6 has enrolled thousands of participants from across the USA and continues to test the effectiveness of repurposed medicines for treating COVID-19. Our lessons learned contribute to the emerging understanding of how to optimize decentralized trials.

## Introduction

### Background

The clinical disease related to severe acute respiratory syndrome coronavirus 2 (SARS-CoV-2) infection, referred to as Coronavirus Disease 2019 (COVID-19), had resulted in approximately 676,609,955 confirmed cases and 6,881,955 deaths worldwide as of May 2023 [1]. Although severe disease has been curtailed except among the most vulnerable, the virus continues to cause infection and disease despite social distancing and masking measures, vaccination campaigns/requirements, and other public health measures.

At this stage of the pandemic, a plethora of clinical trials have been reported, and some medicines have been shown to improve clinical disease and reduce morbidity and mortality [2–4]. Various medicines have been authorized or approved by the U.S. Food and Drug Administration (FDA) for treatment of COVID-19 in the inpatient and outpatient setting [5]. Multiple immunomodulatory agents are being used off-label for the treatment of severe COVID-19 in the inpatient setting [6]. These medicines are recommended only for certain subgroups of patients with COVID-19, and there are few current therapies that can be taken at home. Two medicines, nirmatrelvir-ritonavir (Paxlovid) and molnupiravir, have been shown to have benefit, but uptake has been low perhaps due to concerns about drug–drug interactions, side effects, access to treatment, and other factors. Alternatives are essential to achieve critical goals of helping patients in the outpatient setting feel better faster and preventing disease progression.

Among the compendium of medicines approved by the FDA for medical care in the USA are numerous agents that can be readily self-administered in the outpatient setting, some of which are postulated to be effective for treating COVID-19. Evidence establishing effectiveness of repurposed medicines is critical to promote their use and so that ineffective therapies are not embraced; all medicines have risks and costs associated with them. The need, therefore, is for studies that can establish the effectiveness and confirm the safety of therapies being repurposed to treat COVID-19, specifically those therapies that can be self-administered by the general population in the outpatient setting. We posit that testing the effectiveness of repurposed medicines for the treatment of mild-to-moderate COVID-19 in the outpatient setting can be optimized using a decentralized clinical trial framework.

In April 2020, the National Institutes of Health (NIH) established the Accelerating COVID-19 Therapeutic Interventions and Vaccines (ACTIV) partnership between government, industry, and academic researchers to investigate the most promising treatments and vaccines [7,8]. To execute clinical studies that could adequately test the effectiveness of repurposed medicines, ACTIV-6 was established as a decentralized, platform trial. The goal of ACTIV-6 is to evaluate whether repurposed medicines can make participants with mild-to-moderate COVID-19 who are treated in the outpatient setting feel better faster and reduce death and hospitalization. The trial was designed for a wide range of settings within healthcare systems and the community, including using direct-to-participant procedures, in order to extend reach beyond the traditional academic centers and to maximize generalizability and uptake of treatments found to be effective. Medicines tested in the platform are selected through an external agent prioritization process that considers supporting efficacy data, the established safety record in humans, and whether the agent can be self-administered in the outpatient setting [9]. Through this description of ACTIV-6 and discussion of the design features that make it fit for purpose, we offer insights into the opportunities and challenges for decentralized trials in both pandemic and non-pandemic settings.

## Methods

### Study Design

ACTIV-6 is a multicenter, double-blind, placebo-controlled, randomized, platform clinical trial designed to evaluate the effectiveness of repurposed medicines in reducing symptoms and preventing disease progression in non-hospitalized participants with mild-to-moderate COVID-19. The platform trial can evaluate multiple study drugs simultaneously; within each study drug arm both active study drug and a matched placebo are used. Study drug arms can be added or dropped as the platform progresses. The goal is to have maximum flexibility around the inclusion of repurposed study drugs.

ACTIV-6 is designed to be conducted remotely with enrollment conducted at a central location and through sites across the USA. Symptomatic adults aged ≥ 30 years with a confirmed positive SARS-CoV-2 infection sign up for participation and provide inclusion and exclusion information. Those aged less than 30 were excluded due to the low risk of disease progression in these individuals. Eligible participants are randomized, and study drug is then shipped to a participant’s home. Participants complete regular patient-reported outcomes (PROs) assessments using a web-assisted symptom diary. Treatments are expected to last no longer than 14 days, and participants are on study for up to 180 days.

### Setting and Roles of the Sites

The ACTIV-6 platform was constructed to support fully remote trial activities, fully site-based research activities, and a hybrid where some activities are conducted at a site and others are direct-to-participant. Participating sites include academic medical centers from the Patient-Centered Clinical Research Network, National Center for Advancing Translational Sciences (NCATS) Clinical and Translational Science Awards Program, SignalPath, and Conduct Clinical Trials networks, as well as other regional sites. A full list of site locations can be found in ClinicalTrials.gov (NCT04885530).

Site responsibilities in this trial generally include recruitment, consent, participant management, and serving as a resource for questions about study participation. A call center is available to provide site functions when a participant signs up directly, which is done by following a website link to a REDCap-based intake survey for expression of interest. The call center reviews the interested participant’s information and attempts to link the participant to a local site. If the site does not have capacity or there is no local site, the call center will directly recruit, consent, and manage the participant. The study is also supported by a central pharmacy from which study drugs are shipped, a data coordinating center, and a clinical coordinating center.

### Electronic Data Capture System

ACTIV-6 is a technology-enabled decentralized trial. The REDCap-based electronic data capture (EDC) system facilitates real-time transfer of information between the participants, sites, call center, pharmacy, coordinating centers, and ancillary data systems. A participant’s experience with the EDC begins with an eConsent process; upload of a positive SARS-CoV-2 test using a smartphone, tablet, or laptop camera or file transfer; and completion of baseline medical history and symptoms. Subsequently, the participant receives regular texts or e-mails inviting them to provide adherence, outcome, and safety data; the majority of data in the trial are participant reported. All participant-facing EDC interfaces are available in both English and Spanish.

As information is entered, events can be triggered based on the values reported. Events can include electronic actions such as initiating the randomization process for an individual patient, or they can be messages and alerts. Examples of messages and alerts include: notification to the managing site or call center when a patient is non-adherent with taking study drugs, when new health events or worsening symptoms require a safety review, or to warn about missed data collection. To achieve real-time communication, REDCap is partnered with a customized communications platform that directs the pertinent message to the right user through the Twilio platform (www.twilio.com). The communications platform is hosted within the data coordinating center and includes a reporting bookshelf and the randomization system. The EDC system integrates all available data sources including a clinical trial management system that houses the key study personnel lists; the event adjudication systems; and the courier’s shipment tracking system to verify when study drug arrived.

With a real-time data capture and data-integration system, ACTIV-6 delivers daily accrual and data quality reports, including all of the necessary operational information at the study, site, and participant level for managing the recruitment process, managing participants while on study, and optimizing data quality in real time. While this is a critical design feature for a platform trial producing evidence on a timeline relevant to a pandemic, we posit that a well-designed electronic research record system is essential for decentralized trials where communication among trial personnel and participants is critical.

### Study Population and Enrollment Process

Participants are identified by participating sites or they self-identify via the study website or study hotline(s). Recruitment strategies include outreach through local health systems, pharmacies, and community testing programs, as well as targeted advertising on radio and internet platforms. Recruitment materials include a flyer, information brochure, the study website (activ6study.org), and other websites that have hosted informative videos from physicians such as combatcovid.hhs.gov. A Stakeholder Advisory Committee made up of patients, caregivers, clinicians, and stakeholders who experienced, advocated for, or treated patients with COVID-19 has been engaged throughout ACTIV-6 to provide input on recruitment, patient-facing study materials, and outreach, as described elsewhere [10]. All recruitment materials have received Institutional Review Board (IRB) approval and are available in English and Spanish.

**Figure 1.**
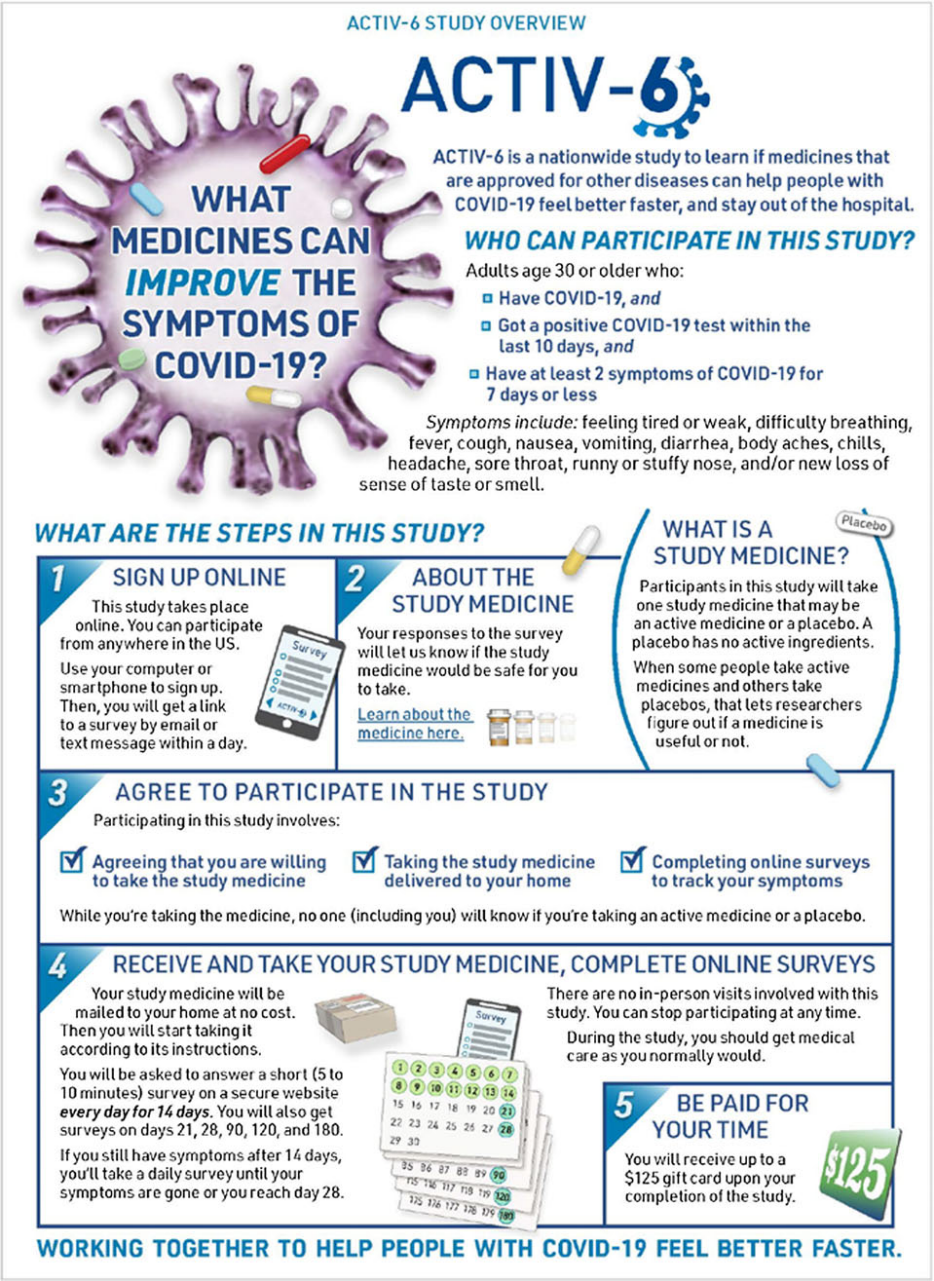
ACTIV-6 study overview graphic. At sites’ discretion, eligible participants who wish to enroll in ACTIV-6 are presented with an informational graphic that provides an overview of the study as a part of the electronic consent process.

**Figure 2.**
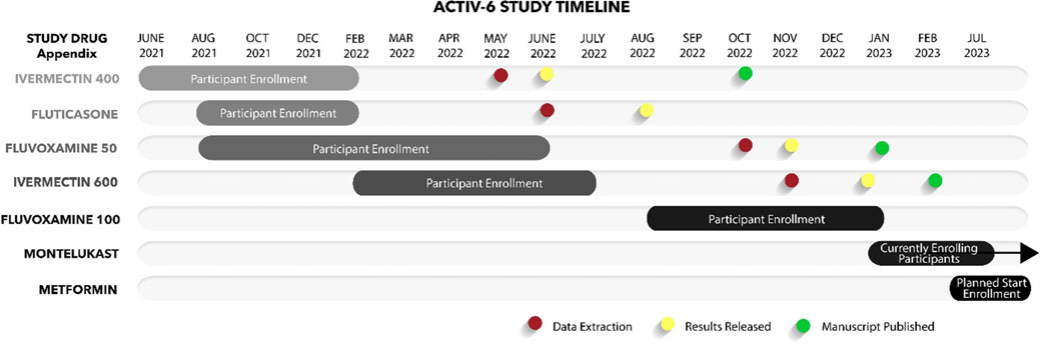
ACTIV-6 study timeline.

To initiate enrollment in the trial, potential participants or their managing site complete an expression of interest that includes contact information and indicates the person is aged ≥ 30 years, has tested positive for SARS-CoV-2 within the past 10 days, and has had at least two symptoms for ≤ 7 days. Participants who self-identify are triaged by the call center to the closest study site or, if the site is unavailable, the call center may manage the participant directly. Participants were preferentially linked to a local site to distribute the recruitment workload, to actively engage sites in the study, and to ensure the continuation of any previously established patient–provider relationships. Once the participant is verified by the site or call center, they complete an electronic consent process. The site or the call center can walk the participant through the process, or the participant may continue in the study as a virtual participant interacting solely through the electronic research record in REDCap. At sites’ discretion, participants are first presented with an informational graphic that provides an overview of the study (Fig. 1), and subsequently all participants are presented with a consent form that follows a traditional format. Participants first review the study as a whole and then the individual study drugs available for testing (Fig. 2). Participants are asked to consent to those study drugs they are willing to try. This complexity was integrated due to expected participant preferences around therapeutic options, such as ivermectin and fluvoxamine, and the desire to give participants the ability to opt in or to opt out of any study agent.

Despite the use of an electronic consent framework, delivering the right consent document has proven complex. Since sites are involved in recruiting and managing participants, the sites must agree to rely on the central IRB and appropriate local context must be incorporated into the informed consent document presented to the participant managed by that site. With over 100 sites and delivery of consent forms in both English and Spanish, the number of documents is high. With new agents being added to and dropped from the platform, the number of documents escalates. The complexity is advanced by different timelines for local review of changes and context. The communications platform is critical to selecting the right consent for delivery to any participant based on site, preferred language, and study arms available at that site.

On completion of consent, participants are first required to upload evidence of any authorized or approved reverse transcription polymerase chain reaction (RT-PCR) or antigen SARS-CoV-2 test collected within 10 days of screening and to document their inclusion and exclusion criteria and onset of symptoms. Symptoms of acute infection must have been ≤ 7 days prior to enrollment and can include fatigue, dyspnea, fever, cough, nausea, vomiting, diarrhea, body aches, chills, headache, sore throat, nasal symptoms, and new loss of sense of taste or smell. Exclusion criteria are current or recent (within 10 days of screening) hospitalization for COVID-19 infection; current or recent use (within the last 14 days) of study drug; and any known sensitivity, allergy, or contraindication to the study drug. Additional baseline data collected immediately after consent include contact information for study drug and survey delivery and baseline quality of life information. Prior to proceeding to randomization, personnel at the site or the call center review the participant information to confirm eligibility, including review of the uploaded SARS-CoV-2 test result. This step of reviewing source documentation confirming eligibility reflects the evolving nature of roles and responsibilities when trials are performed in a decentralized manner.

### Randomization

**Figure 3.**
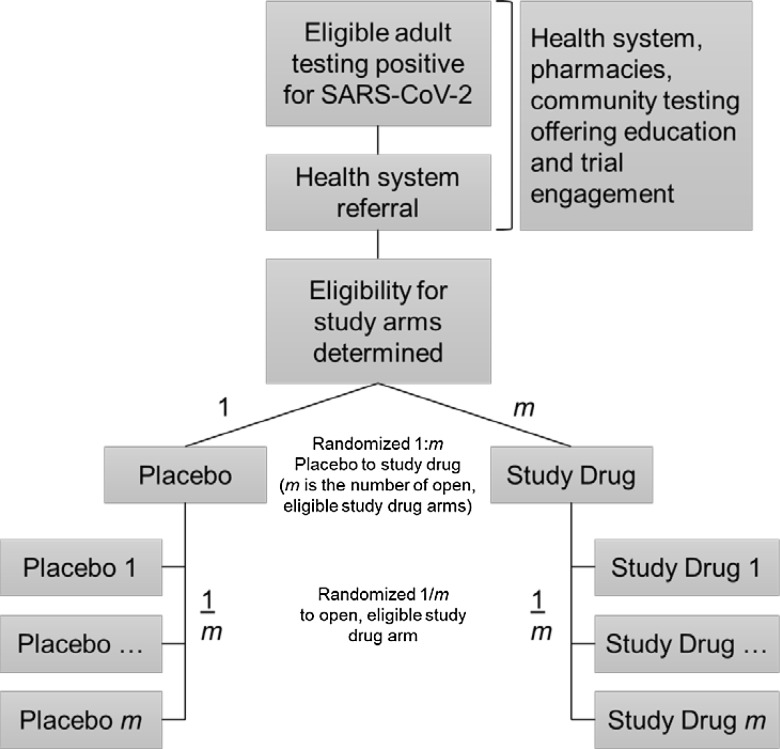
Recruitment and two-step randomization procedure for eligible participants enrolling in ACTIV-6.

Participants must be eligible for, and consent to, at least one study drug arm in order to proceed to randomization. To date, up to three study drug arms have been open in parallel, with placebo participants shared among them. To achieve a balanced number of participants randomized to placebo or active agent within each study drug arm, a two-step randomization process is used (Fig. 3). At the first step, the participant is assigned to receive an active agent or a placebo. The ratio for this allocation is based on the number of study drug arms the participant could enter. If the participant is eligible and consents to only one study drug arm, the allocation is 1:1 (and the procedure terminates). If the participant is eligible and consents for two study arms, then the allocation is 1:2 placebo to active agent. The participants randomized to active agent are subsequently randomized with equal probability to which active agent they receive, and the placebo participants are randomized with equal probability to which of the two active agents they will be matched. This maximizes the number of participants exposed to a potentially beneficial active agent and minimizes the overall sample size because the placebo participant contributes to both study arms. A natural consequence is having both matched placebos and unmatched contributing placebos in the comparator group for any study drug.

### Blinding

ACTIV-6 is a blinded trial. The investigators, treating clinicians, statisticians, and study participants are aware of which study drug arm the participant is randomized to, but not whether they are in receipt of active agent or placebo. Only the pharmacy staff who are handling randomization codes and unblinded members of the data coordinating center team are unblinded. At the request of a treating clinician, and only with approval from the study’s medical monitor, unblinding can occur if required for participant safety or treatment. Unblinding is facilitated and tracked by the EDC.

### Study Interventions

ACTIV-6 is designed to evaluate the effectiveness of repurposed medicines against the background of prevailing care standards for COVID-19. Study drugs are selected based on recommendations from the ACTIV Agent Selection Committee sponsored by the Foundation for the National Institutes of Health [9]. The specific study drugs are not described in detail here; more information can be found in publications describing the results of ACTIV-6 [11–14]. Briefly, for inclusion in the platform a study drug arm is described in a protocol appendix. The appendix describes drug-specific exclusion criteria, drug-specific procedures, details of the matching placebo, and other drug-specific information. At the time of this writing, the study drug appendices include ivermectin (two doses studied), fluvoxamine maleate (two doses studied), inhaled fluticasone furoate, montelukast, and metformin.

After consent and randomization, the central pharmacy prepares a complete study drug packet for distribution to the participant by overnight shipping. Packaging is labeled to indicate that the product is for investigational use. Taking advantage of the decentralized nature of the trial, two different pharmacies have been used to meet the high demands of packaging and distributing study drug and placebo. Delivery of study drug is tracked through shipping logs from the courier as well as participant notification of drug receipt. Use of study drug is tracked via the EDC, call center, or sites. Participants are expected to dispose of any unused study drug as they would normally when stopping a medicine.

#### Schedule of events

Once enrolled, participants progress through the trial by taking their study drug and completing electronic patient-reported outcome assessments (ePROs), as shown in Table 1. To account for potential delays between randomization and start of study drug, “study day 1” is defined as the day when study drug is delivered to the participant. Daily assessments take place from study days 1 through 14. For participants who do not report three consecutive days of no symptoms, daily assessments for symptom burden continue through study day 28 or until symptoms resolve for≥3 consecutive days. Follow-up occurs for all participants on study days 21, 28, and 90, at which time participants are prompted to complete an ePRO. Given growing interest in long COVID, follow-up was extended to 120 days for participants enrolling after August 25, 2022. For most participants enrolling after February 13, 2023, the final visit occurs at 180 days. Participants are followed until their final follow-up, withdrawal of consent, or death. A participant may withdraw from the study at any time at his/her own request, or may be withdrawn at any time at the discretion of the investigator for safety, behavioral, compliance, or administrative reasons. Those who wish to withdraw from taking their study drug before the protocol-defined duration are asked to continue with data collection through the final visit.

**Table 1.** Schedule of events for participants enrolled in ACTIV-6

	Screening	Intervention period		Follow-up period						Final visit	Unplanned study visit
Day	Within 2 days of Day 1	Day 1	Days 2–14	Day 15–20	Day 21 ± 2	Day 22–27	Day 28 + 5	Day 90^1^ + 5	Day 120**^1^** + 5	Day 180^1^ + 7	
ACTIV-6 trial											
Consent	X										
Demographic information	X										
Eligibility criteria confirmed	X										
Randomization	X										
Receipt of study drug or placebo		X									
Continued use study drug		Continuous^2^									
Clinical assessments											
Abbreviated medical history	X										
Self-reported pregnancy	X^3^										
Concomitant therapy	X	X^4^	X^4^								
Remote visit			X^5^				X				
Drug Adherence		X	X								
COVID-19 outcomes		X	X^9^		X		X	X	X	X	
Symptom reporting	X^6^	X	X^6^	X^6^	X^6^	X^6^	X^6^	X	X	X	
PASC symptom questionnaire										X	
QOL questionnaire	X		X^7^				X	X	X	X	
At-home pulse oximetry			X^8^								
Columbia-Suicide Severity Rating Scale (C-SSRS)			X^9^								
Safety assessment^10^		Continuous via online system and medical record review^10^									X

1 Day 180 is applicable only for participants who were consented after protocol v7.0 was implemented; day 120 is applicable only for participants consented on protocol v6.0; day 90 was the final follow-up day for all other participants.

2 Refer to study drug details above for length of study drug administration.

3 Only for enrollment in Study Drug Appendices that have pregnancy listed as a contraindication for females of childbearing potential. Participants will self-report pregnancy using the Pregnancy Reasonably Excluded Guide.

4 Review only during study drug/placebo administration if contraindicated medications provided for the study drug arm, per Appendix.

5 Day 14 only.

6 Daily symptom reporting; continued daily beyond day 14 through day 28 until symptoms resolve for≥3 consecutive days. All participants will complete symptom reporting on days 21 and 28, regardless of symptom resolution.

7 Day 7 and 14 only.

8 Day 3, 7, and 14 only.

9 At day 7 and 14 for participants enrolled in Appendix E – Fluvoxamine Maleate 100.

10 Participant’s medical record will be reviewed to confirm serious adverse events (SAEs), unanticipated adverse device events (UADEs) (as applicable), and events of special interest (ESIs).

Given that all data collection is via electronically delivered PRO tools, it is expected that some data will be missing. The sites or call center are instructed to contact participants to complete the information whenever possible. A participant who chooses not to respond on a given day is not classified as withdrawn.

### Outcomes

A unique feature of this trial is that the primary outcome is selected between time to sustained recovery (within 28 days) or hospitalization or death (within 28 days) with the choice made immediately preceding each analysis. The choice is recommended by the Executive Committee, who are blinded to results. The recommendation is reviewed by an independent data monitoring committee (IDMC), and the decision is vetted by the trial oversight committees. Making the selection in this way allows the study results to reflect the pandemic context during the applicable enrollment period. Early in the pandemic, high rates of mortality and hospitalization would suggest the feasibility of evaluating the effect of study drugs on preventing disease progression by measuring clinical event rates. Later in the pandemic, effects of study drug on improvements in symptoms have become the priority. The possibility of a new variant resulting in more severe disease remains so clinical events may become a relevant outcome once again.

For this platform, time to sustained recovery is defined consistent with the FDA guidance on COVID-19 clinical trials [15] and occurs when the participant reports the third of 3 consecutive days without COVID-19 symptoms. To assess symptoms, participants were asked whether their COVID-19 symptoms were being experienced as none, mild, moderate, or severe *that day*. If symptoms were observed, each of the following potential symptoms was queried: fatigue, dyspnea, fever, cough, nausea, vomiting, diarrhea, body aches, sore throat, headache, chills, nasal symptoms, new loss of sense of taste or smell, or other COVID-related symptom.

Multiple secondary outcomes are considered. A model-based estimand was chosen to describe the clinical effect of the therapy: mean time unwell. This is an estimate of the amount of time participants spend in an undesirable health state. Unlike time to recovery, the mean time unwell naturally accommodates mortality as an undesirable health state. Additional secondary outcomes were hospitalization or death at days 14 and 28 (if not primary); time to recovery (if not primary); mortality through day 28; hospitalization, urgent care, emergency room visit, or death through day 28; COVID Clinical Progression Scale [16] at days 7, 14, and 28; and Patient-Reported Outcomes Measurement Information System (PROMIS)-29 [17] on days 7, 14, 28, and 90. Safety outcomes include all potentially associated adverse events as well as any events of interest defined for the individual study drug arms.

### Data Collection, Monitoring, and Dissemination

Taking advantage of the direct-to-participant nature of this trial, the majority of data are reported by the participants directly using electronic surveys. Participants are sent links to online surveys via text message or email. Data collection instruments are provided in both English and Spanish. Designated study personnel may also conduct study assessments in-person or by phone and enter the corresponding data directly. Medical records are accessed to provide information about healthcare utilization and adverse events as needed. Data quality is reviewed using logic checks at the time of data entry and an embedded query process.

It is notable that in a decentralized trial where data are reported directly by participants, the process of data cleaning and monitoring differs from traditional trials. Daily data quality reports provide the clinical coordinating center with information about missing and inconsistent data elements, or where source documents might be needed to verify healthcare utilization events. When participants do not complete their surveys, sites are notified to follow-up and can fill in data verbally reported by participants over the phone. On completion of a study arm, the process for cleaning the data is focused on validating clinical events and eligibility. The EDC is the sole source for ePROs so any residual error remains in the dataset. Systematic data quality rules are applied to prevent implausible data in analytical datasets.

### Statistical Analysis

This platform trial is designed to add and remove study drugs over time, with the use of shared placebos among overlapping study drug arms. Once 28-day outcome collection is completed for all contributors to a study drug arm, a dataset inclusive of all persons on active treatment and both matched and contributing placebos is extracted and validated. It is expected that as data continue to accrue in the platform, there may be changes to some information for participants contributing to an analysis, such as discovery of a clinical event after the data extract occurs. Therefore, the dataset as it existed for the analysis is locked and archived for reproducibility. The data are also pseudonymized so that the blinded statisticians are unable to reasonably link the analytic dataset back to the EDC.

For each study drug, inferences about the effect of active study drug versus placebo are made primarily using covariate-adjusted Bayesian regression methods. A modified intention to treat approach is used; all participants who receive study drug are included as assigned, and any participant who does not receive study drug (failure of delivery, participant death, or participant withdrawal prior to receipt of study drug) is excluded from analysis. All available data are used regardless of post-randomization adherence to study protocols. While response rate and patterns of survey response are an area of methodological exploration, for primary analyses participants contribute if they complete at least one ePRO after baseline. The statistical analysis plan may be customized for individual study drug arms. For example, for one study drug arm emergency department and urgent care visits have been included in the healthcare utilization outcome for the futility assessment since emerging data suggest the potential of drug effects on this composite outcome.

#### Interim analyses

Interim analyses are planned at *N* = 300, 600, and 900 participants meeting criteria for inclusion in a modified intent to treat cohort for any study arm, with analysis after day 28 endpoints are collected. The interim reports are provided to the IDMC, which include several quantities related to the efficacy and safety of the intervention. Guidance quantities for decision making were established *a priori*. For futility, a posterior predictive probability of efficacy at *N* = 1200 of < 0.05 was set. For efficacy, a posterior probability of efficacy on the primary endpoint of > 0.95 was set.

#### Primary analysis

The primary effect of study drug versus placebo is quantified using either occurrence of clinical events (hospitalization or death) or time to recovery. Clinical events use a Bayesian logistic regression model with the difference in hospitalization or death rates summarized as a treatment effect odds ratio. For time to recovery, the treatment effect is estimated from a proportional hazards regression model of the recovery endpoint in which participants who die are retained in the risk set, to account for the competing risk of mortality.

The regression model includes the following covariates: age as a restricted cubic spline with 3 knots; self-reported gender; duration of symptoms prior to treatment; calendar time as restricted cubic spline with 4 knots; vaccination status (full and partial vs none); geographic region; call center indicator; and additional appendix-specific covariates relevant to baseline disease severity and patient risk. The regression parameter for randomization assignment is assigned a normal prior distribution with mean zero and standard deviation 0.1 selected so that type I error is bounded within 0.05. All other regression parameters have a weakly informative prior. The standard suite of model diagnostics for Bayesian models is implemented, including graphical and analytical checks of the adequacy of the posterior samples, the model specification, and its predictions.

#### Secondary endpoint analyses

Table 2 summarizes the estimands and analysis methods for each secondary endpoint. Briefly, the COVID Clinical Progression Scale score on days 7, 14, and 28, and the modified PROMIS-29 score on days 7, 14, 28, and 90 are compared between participants in each study drug arm versus the placebo arm using a covariate-adjusted cumulative probability ordinal regression with logit link using weakly informative priors. For estimating time to event endpoints (e.g., time to death, hospitalization, urgent care, or emergency room visit), a Cox proportional hazards regression with weakly informative priors is used. Mean time unwell for each individual treatment arm is estimated from the primary endpoint model.

**Table 2.** Statistical approaches for ACTIV-6 secondary endpoints

Endpoint	Estimand	Analysis method
Mean time unwell	Difference in means	Longitudinal ordinal regression model
Hospitalization or death (day 14 and day 28)	Model-based estimates of the treatment effect odds ratio*	Logistic regression
Mortality (day 28)	Model-based estimates of the treatment effect odds ratio*.	Logistic regression
Mortality (time to event)	Model-based estimates of the treatment effect hazard ratio*	Cox regression
Hospitalization, urgent care, emergency room visit, or death (time to event within 28 days)	Model-based estimates of the treatment effect hazard ratio*	Cox regression
COVID Clinical Progression Scale (day 7, day 14, and day 28)	Treatment effect odds ratio	Cumulative probability ordinal regression with logit link
Modified Patient-Reported Outcomes Measurement Information System (PROMIS)- 29 (day 7, day 14, day 28, and day 90)	Treatment effect odds ratio	Cumulative probability ordinal regression with logit link`

* If the number of events is less than 30, a descriptive analysis is performed.

#### Heterogeneity of treatment effect

Analysis of differences in treatment efficacy as a function of pre-existing participant characteristics, also referred to as heterogeneity of treatment effect, is pre-specified for: vaccination status; calendar time (to reflect the changing pandemic context) as a restricted cubic spline with 3 knots; duration of symptoms (time from symptom onset) as a restricted cubic spline with 3 knots; age as a restricted cubic spline with 3 knots; body mass index as a restricted cubic spline with 3 knots; and patient-reported symptom severity (none, mild, moderate, severe). Heterogeneity of treatment effect is assessed by estimating model posterior probabilities for a full model including interaction term(s) with randomization assignment and a reduced model without the interaction term(s).

#### Missing data

Missing data in the covariates is handled with imputation and posterior stacking. If the percentage of observations with missing covariates exceeds 5%, then multiple imputation (predictive mean matching) is used. Otherwise, single imputation (conditional mean of the complete cases) is used.

#### Sample size determination

The platform was originally intended to enroll up to about 15,000 adults and to include up to seven study drug arms. For an initial estimate of sample size, it was determined that approximately 1200 participants per study drug arm would be sufficient to conclude whether there is meaningful evidence of benefit defined as a reduction in symptom duration of more than 1 day; this was based on crude estimates that have since been replaced with more comprehensive simulation-based estimates of power.

Simulations were performed on Amazon Elastic Compute Cloud servers using the statistical computing software, R (4.1.2). In each replicate, pseudo trial data were generated to mimic the type of data anticipated in the trial, and then it was analyzed according to the primary endpoint analysis plan described above, except for the covariate adjustment. To understand the impact of the planned interim analyses, each pseudo dataset was analyzed using 300, 600, 900, and 1200 participants. The distribution for time to sustained recovery used in the simulations was selected, so that 88% of placebo participants experienced recovery within 28 days, a rate that had been observed in the placebo arm of other outpatient clinical trials of COVID-19 at the time of calculation.

The first task was to determine the prior variance of treatment effect so that the family-wise false-positive error rate was < 0.05. This was achieved by generating pseudo data in which recovery times of the active arm matched the distribution of the placebo counterparts. If a pseudo dataset resulted in a determination of efficacy at any N, the conclusion was deemed an error. The proportion of errors among 5000 pseudo datasets was calculated for each choice of prior variance. The prior variance was reduced until the error rate was < 0.05, in this case 0.01.

The second task was to determine the power with the prior variance determined in the first task. Like before, pseudo datasets were created, only this time the distribution of the recovery time in the active arm was shifted (on the log relative hazard scale) to reflect improved recovery times and a treatment benefit. Now, if a pseudo dataset resulted in a determination of efficacy at any N, the conclusion was deemed correct. The proportion of correct conclusions among 5000 pseudo datasets was calculated for increasingly larger treatment effects. For a treatment effect hazard ratio of 1.17, 80% of pseudo datasets concluded efficacy.

### Trial Oversight

ACTIV-6 is funded through special COVID-19 appropriations such as the American Rescue Plan Act and is managed by the NCATS, one of the 27 NIH institutes and centers. NCATS and the ACTIV public-private partnership are responsible for the overall stewardship of ACTIV-6. Oversight of master protocol design, agent prioritization, and overall direction/scope of the trial are overseen by NCATS and several committees including an ACTIV-2, ACTIV-3, and ACTIV-6 Trial Oversight Committee that are associated with the ACTIV Therapeutics Clinical Working Group. ACTIV Steering Committee oversees the trial operations and progress. Members include representatives from clinical sites, trial coordinating centers, NIH, PCORI, H-CORE (originally Operation Warp Speed), FDA, NCATS, ACTIV representatives with no conflict of interest, and academic and industry subject matter experts. Duke Clinical Research Institute (Durham, NC) serves as the clinical coordinating center and the call center and is responsible for study coordination, site management, clinical event adjudication and safety monitoring, data monitoring and cleaning, communication, and financial administration. Vanderbilt University Medical Center (Nashville, TN) serves as the data coordinating center and is responsible for treatment allocations, receipt and processing of data, quality control programs, and statistical analysis and reporting. WCG IRB provides US Central IRB oversight.

An IDMC oversees the safety and welfare of trial participants as well as provides recommendations for continuation, discontinuation, or revision of the trial. In addition to routine evaluation of decision thresholds, regular IDMC reviews are conducted to monitor recruitment progress; participant enrollment; adherence, retention, and status of data collection; events of special interest; unanticipated problems; and serious adverse events. In addition, selection of endpoints at the time of analysis is reviewed by the IDMC, as is the addition or stopping of study drug arms.

Treatment with repurposed medicines in this trial is through an investigational new drug (number 155481) application submitted to the FDA. The trial was registered with ClinicalTrials.gov (NCT04885530) prior to enrollment of the first participant on June 8, 2021. This trial is conducted in compliance with the International Council for Harmonization E6 (R2) guideline for Good Clinical Practice and the applicable regulatory requirements from the United States Code of Federal Regulations (CFR), including 45 CFR 46 (Human Subjects Protection); 21 CFR 312 (Investigational New Drug); 21 CFR 50 (Informed Consent), and 21 CFR 56 (IRB).

### Trial Adaptations

ACTIV-6 was designed to be highly adaptive; the dynamic nature of a pandemic highlights the need for clinical trial design to allow for changes in the context of a disease. The ACTIV-6 study opened in June 2021 with a study drug arm investigating ivermectin at a dose of 400 µg/kg. As new evidence emerged [18–20], it was considered necessary to investigate ivermectin at an increased dose. In February 2022, the ivermectin study drug arm at 400 µg/kg completed enrollment and a new study drug appendix investigating ivermectin at 600 µg/kg was added with no disruptions to trial enrollment. Vaccination status was added as a data element in early 2022. During initial protocol development, vaccinations were not yet widely available, they were controversial, and vaccination rates among the trial’s target population were low [21]. As vaccination campaigns and requirements ramped up and new evidence emerged of breakthrough cases in vaccinated adults [22], vaccination information was added. Finally, as new evidence emerged on the long-term effects of COVID-19, follow-up visits were added first for day 120 and subsequently for day 180 to investigate the potential of acute treatments to limit or prevent post-acute sequelae of COVID-19 (PASC), also known as long COVID [23].

### Dissemination

The results generated from ACTIV-6 are being disseminated to the public and the medical community through presentations at scientific meetings and publishing manuscripts in peer-reviewed journals. Broad dissemination also occurs through the same portals used to recruit participants. Participant-level data will be made available to qualified investigators at the end of the platform trial by archiving a fully de-identified dataset in a public repository such as the National Heart, Lung, and Blood Institute’s BioData Catalyst. The results are also returned to participants. Study summaries are generated with input from lay stakeholders and are posted publicly on the ACTIV-6 study website. A notification on the availability of results is distributed to enrolled participants leveraging the direct-to-participant communication framework. The complete role of the stakeholder committee in ACTIV-6 is detailed elsewhere.

#### Current status

The ACTIV-6 study opened on June 8, 2021 with the ivermectin study drug appendix and dosing at 400 µg/kg. Fluticasone furoate and fluvoxamine maleate 50 mg study drug appendices were added to the platform on August 6, 2021. Ivermectin at 400 µg/kg, fluticasone furoate, and fluvoxamine maleate 50 mg completed enrollment on February 4, February 8, and May 27, 2022, respectively. The ivermectin appendix at a 600 µg/kg dose opened on February 16, 2022 and completed enrollment on July 22, 2022. After a brief pause in enrollment, the ACTIV-6 study re-opened with fluvoxamine maleate at 100 mg on August 25, 2022, which completed enrollment on January 20, 2023. The montelukast appendix was open for enrollment from January 27, 2023, to June 23, 2023. The metformin arm opened for enrollment on September 6, 2023. ACTIV-6 is currently enrolling under protocol version 10.0 dated August 2, 2023, with trial completion expected by June 30, 2024.

## Discussion

Progress has been made toward understanding the pathology of SARS-CoV-2 and identifying treatment options for COVID-19. Much work remains to identify outpatient therapies that improve symptoms and reduce progression to severe disease in both vaccinated and unvaccinated populations. The ACTIV-6 platform was designed to generate this evidence for repurposed medicines in the outpatient treatment of COVID-19. A decentralized trial approach was selected as being critical to support rapid enrollment of a diverse outpatient population reflective of those experiencing COVID-19, removing many physical barriers to trial participation. Much has been learned about the decentralized approach.

In order to meet the demands of the trial in a low-touch way, trial recruitment, informed consent, and data collection and monitoring all occur remotely. Recruitment occurs nationally through enrolling sites, online and radio advertising, and outreach through local health systems and community testing centers. The creation of an electronic research record that functions as the primary interface between participant, sites, call center, pharmacy, and coordinating centers allows for remote electronic informed consent, regular outcome assessments directly from participants, and provides a way for coordinators to perform remote safety and data quality monitoring.

At study outset, there was much discussion about the role of sites. The regulatory framework of requiring IRB reliance, local regulatory requirements, and addition of local context to a consent form can be a major barrier to multisite research that decentralized activities could overcome, but only if the role of sites is restricted to activities that are considered not engaged in research, such as participant identification and referral. Yet, recruiting and engaging participants are a major reason to retain a role for sites. ACTIV-6 chose to accept some degree of regulatory burden (present for about 50% of sites, mostly academic medical centers and faith-based hospital systems) in return for having sites taking a more active role in recruiting and managing participants. If single IRB review and oversight were truly effective, future studies could benefit from having both streamlined regulatory processes and site-based management of participants. There remains much opportunity to clarify regulatory oversight of trials conducted in a decentralized manner.

Unanticipated problems have included participants signing up repeatedly, which we have tried to prevent using name matching algorithms and verification. Some participants consented to multiple study drug arms but then withdrew when not randomized to their preferred study drug arm, and some participants chose not to respond to surveys; our statistical methods were designed to include as much information as a participant was willing to provide. An important lesson learned is related to disparities in courier and delivery systems. While we used a decentralized approach to maximize access for persons living anywhere in the USA, couriers do not always deliver directly to the recipient’s door and the timeline for delivery could make participants ineligible by the time study drug arrived. The increasing use of centralized pick-up locations for packages instead of door-to-door delivery also inhibits proper drug delivery. While decentralized trials can improve access and rapid accrual, careful attention to factors not usually of concern to researchers become important. When taking the trial to the participant, it is critical to understand the participant’s lived context and barriers to serving the participant in their setting.

There are additional strengths and novelties of the ACTIV-6 design. We developed a decentralized approach to trial recruitment, informed consent, and data collection and monitoring. Recruitment occurs using a variety of methods designed to reach a widespread and diverse patient population. Purposefully allowing for in-person, telephone, and electronic approaches to enrollment was intended to maximize opportunities for participant identification and to remove recruitment barriers and we were able to successfully enroll participants in every state. While not a focus of this article, the ACTIV-6 investigators are using information on source of recruitment to explore the yield of various approaches as well as factors associated with improved recruitment. The use of REDCap allows for a secure and robust electronic system to capture informed consent and baseline, daily assessment, and outcome data directly from a patient, while also allowing a way for coordinators to perform remote safety and data quality monitoring. Formal evaluations of the effectiveness of these systems are currently underway.

In summary, ACTIV-6 has enrolled thousands of participants to test repurposed medicines for improving symptoms and preventing disease progression in COVID-19. Using a decentralized approach, participants have been successfully enrolled from across the USA reflecting optimization of the design for accrual rates and reach. The platform has been able to adapt to context with additional data collection and extension of follow-up windows. We have shown lack of effectiveness of ivermectin, fluvoxamine, and fluticasone furoate at the studied doses, and the platform continues to accrue participants to test other proposed medicines for COVID-19.

## Supporting information

The Accelerating Covid-19 Therapeutic Interventions and Vaccines (ACTIV)-6 Study Group supplementary material The Accelerating Covid-19 Therapeutic Interventions and Vaccines (ACTIV)-6 Study Group supplementary material

## Supplementary material

The supplementary material for this article can be found at https://doi.org/10.1017/cts.2023.644.
